# Mechanisms of Life Span Extension by Rapamycin in the Fruit Fly *Drosophila melanogaster*

**DOI:** 10.1016/j.cmet.2009.11.010

**Published:** 2010-01-06

**Authors:** Ivana Bjedov, Janne M. Toivonen, Fiona Kerr, Cathy Slack, Jake Jacobson, Andrea Foley, Linda Partridge

**Affiliations:** 1 Institute of Healthy Ageing, Department of Genetics, Evolution and Environment, University College London, Darwin Building, Gower Street, London WC1E 6BT, UK; 2 Max Planck Institute for Biology of Aging, Gleueler Straße 50a, 50931 Köln, Germany

**Keywords:** HUMDISEASE, PROTEINS

## Abstract

The target of rapamycin (TOR) pathway is a major nutrient-sensing pathway that, when genetically downregulated, increases life span in evolutionarily diverse organisms including mammals. The central component of this pathway, TOR kinase, is the target of the inhibitory drug rapamycin, a highly specific and well-described drug approved for human use. We show here that feeding rapamycin to adult *Drosophila* produces the life span extension seen in some TOR mutants. Increase in life span by rapamycin was associated with increased resistance to both starvation and paraquat. Analysis of the underlying mechanisms revealed that rapamycin increased longevity specifically through the TORC1 branch of the TOR pathway, through alterations to both autophagy and translation. Rapamycin could increase life span of weak insulin/Igf signaling (IIS) pathway mutants and of flies with life span maximized by dietary restriction, indicating additional mechanisms.

## Highlights

► Rapamycin, a drug that inhibits TOR pathway, improves longevity in *Drosophila* ► Rapamycin longevity effects are mediated through the TOR pathway ► Life span extension by rapamycin is through translation changes and autophagy ► Rapamycin extends life span beyond dietary restriction and mild IIS mutations

## Introduction

The TOR (target of rapamycin) pathway is a highly conserved nutrient-sensing pathway that functions in such evolutionarily distinct organisms as yeast and mammals to regulate growth and metabolism in response to growth factors, amino acids, various stresses, and changes in cellular energy status. It has also been implicated in the control of protein translation and ribosome biogenesis, upregulation of which is required for growth (for detailed review, see Wullschleger et al., 2006). More recently, the TOR pathway has emerged as an important modulator of aging, an effect firmly established in several invertebrate models (Kaeberlein and Kennedy, 2008).

The central component of the TOR pathway is TOR kinase, which participates in two different multiprotein complexes: TORC1 and TORC2. TORC1 regulates translation and growth through phosphorylation of two key downstream effectors, S6K and 4E-BP. Thus, under favorable conditions, such as an amino acid-rich diet, when TORC1 is active, phosphorylated S6K functions as a positive mediator of the TOR pathway by promoting cellular and organismal growth, and additionally by altering metabolism (Montagne et al., 1999; Um et al., 2006). Accordingly, S6K-deficient animals are smaller, and their metabolism replicates conditions of low-calorie diet, although the exact mechanisms responsible are not completely understood (Um et al., 2006). TORC1-dependent phosphorylation of 4E-BP disrupts its association with the translation initiation factor 4E (eIF4E), allowing the latter to promote cap-dependent translation. All nuclear-encoded eukaryotic mRNAs possess a cap structure consisting of 5′ 7-methylguanosine, which allows them to be recruited to the ribosome and translated in a cap-dependent manner when conditions are favorable (Sonenberg and Hinnebusch, 2009). However, under stress, such as lack of nutrients or growth factors, and in hypoxia, cap-independent translation increases (Sonenberg and Hinnebusch, 2009), allowing a switch to synthesis of other proteins, such as growth factors and heat shock proteins (Baird et al., 2006), which enables the cell to respond to stress and to survive, rather than to initiate apoptosis.

Under starvation, TORC1 additionally promotes autophagy, a critical process for supplying starving cells with bioenergetic components (Lum et al., 2005). During autophagy, cytosolic contents targeted for degradation, including proteins, lipids, nucleic acid, and carbohydrates, are enclosed by a double-membrane structure, the autophagosome, which ultimately fuses with a lysosome for degradation of its cargo (Klionsky, 2007). This not only provides the cell with nutrients and energy but also removes damaged cellular components and thus has an important cytoprotective function.

In contrast to the plethora of known TORC1 functions, the TORC2 complex seems to be mainly involved in actin organization and, additionally, to upregulate the IIS pathway by activating phosphorylation of Akt, the main kinase in the IIS pathway (Wullschleger et al., 2006). Akt can then phosphorylate and inactivate TSC2, a TOR pathway suppressor. Contrary to the positive effects of TORC2 on the IIS pathway, S6K, a downstream component of TORC1, inhibits IIS by negatively regulating the insulin receptor substrate (IRS) at the level of transcription, degradation and phosphorylation (Um et al., 2006; Wullschleger et al., 2006). Thus, there is a complex cross-wiring between the two signaling pathways, the outcome of which may depend on cell type and on the intensity and duration of the signal (Sarbassov et al., 2006).

TOR kinase is inhibited by rapamycin, a natural macrolide compound isolated from bacteria, analogs of which are approved for human use as immunosuppressants and in anticancer therapy (Guertin and Sabatini, 2009; Hartford and Ratain, 2007). Rapamycin is the most specific TOR inhibitor known and inhibits through association with the intracellular protein FKBP12, which then binds to the FKBP12-rapamycin-binding (FRB) domain of TOR, inhibiting TORC1 activity. Although rapamycin does not bind the catalytic domain of TOR, it reduces phosphorylation of two downstream TORC1 targets, S6K and 4E-BP. Rapamycin is generally accepted as an inhibitor of cap-dependent translation, and its capacity to upregulate autophagy and cap-independent translation is well described (Grolleau et al., 2002; Ravikumar et al., 2004).

Inhibition of TOR activity can delay the aging process, as evidenced by increased life span in yeast (Kaeberlein et al., 2007), worms (Hansen et al., 2007; Vellai et al., 2003), flies (Kapahi et al., 2004; Luong et al., 2006), and mice (Selman et al., 2009) with mutations in TOR pathway components. For instance, in yeast, both chronological and replicative life span are extended in the tor1Δ mutant (Kaeberlein et al., 2007) and worms bearing mutations in genes encoding the worm homolog of TOR (let-363) and S6K (rsks-1), and components of the translational machinery, such as ribosomal proteins and translation initiation factors, live longer (Hansen et al., 2007; Kaeberlein and Kennedy, 2008; Syntichaki et al., 2007). Flies mutant for TOR, and with overexpression of dominant-negative forms of S6K or TSC1 or TSC2, which encode negative regulators of TOR, are all long lived (Kapahi et al., 2004; Luong et al., 2006). Recently, it has been shown that genetic deletion of S6K1 in mice extends life span (Selman et al., 2009). Taken together, these data demonstrate an evolutionarily conserved role for TOR in determination of life span.

The TOR pathway may also play an important role in dietary restriction (DR), a key antiaging intervention in which reduced food intake without malnutrition results in extended life span in most organisms so far tested (Bishop and Guarente, 2007; Mair and Dillin, 2008), including rhesus monkeys (Colman et al., 2009). Although the mechanisms underlying DR are not entirely understood, DR does not further increase life span when TOR signaling is already reduced in yeast, worms, or flies, suggesting that common mechanisms may mediate both of these antiaging interventions (Grandison et al., 2009). This may be related to the importance of amino acids for both of these longevity responses (Mair et al., 2005; Sancak and Sabatini, 2009).

To explore mechanisms underlying the role of the TOR pathway during aging, we have taken a pharmacological approach using *Drosophila*, which provides an ideal context for in vivo studies of TOR signaling and aging, because components of this pathway show strong evolutionary conservation from flies to mammals, and *Drosophila* TOR pathway mutants are well-described.

We administered rapamycin to adult flies by feeding and found that it robustly extended life span, exclusively through the TORC1 branch of the pathway. Rapamycin-mediated life span extension was not associated with alterations in either IIS or AMP-activated protein kinase (AMPK) activity but was blocked by alterations to both autophagy and translation. In addition, rapamycin extended life span beyond the maximum seen with DR and lessened the reduction in life span with full feeding, suggesting that rapamycin treatment captures some of the mechanisms by which DR extends life span but also acts through additional mechanisms. Moreover, our results showed that a weak, but not a strong, IIS mutant could extend life span of flies with life span maximized through rapamycin treatment, indicating additional mechanisms. Thus, rapamycin acts at a level in the TOR pathway that captures multiple downstream target processes to extend life span.

## Results

### Rapamycin Treatment Efficiently Reduces Phosphorylation of S6K In Vivo

To determine if rapamycin inhibits TOR activity in vivo in *Drosophila*, we fed rapamycin to flies. To avoid developmental effects, flies were reared on normal food and then given food supplemented with rapamycin from early adulthood (see the Experimental Procedures and the Supplemental Information available online for details of fly stocks and food media). By using a blue-dye feeding assay combined with feeding observations (Wong et al., 2009), we found no significant effect of rapamycin on feeding behavior or on the amount of food consumed (Figure S1). The concentration of rapamycin in flies fed on 200 μM food, measured by mass spectrometry, was 3.3 ± 0.2 ng/mg wet weight, comparable to the effective dose administered to mice for life span analysis (Harrison et al., 2009).

**Figure 1:**
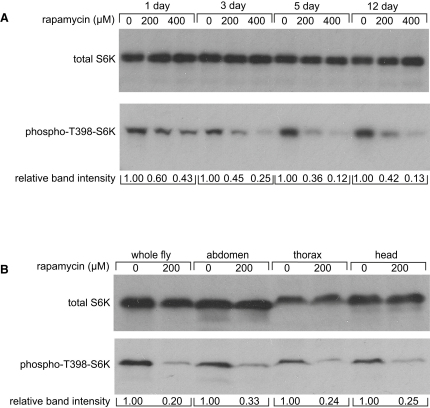
Rapamycin Treatment of Adult *Drosophila* Downregulates TOR Activity (A) Western blot analysis of phospho-S6K on whole-fly protein extracts. Flies were sampled after 1, 3, 5, or 12 days of rapamycin treatment at concentrations of 200 or 400 μM. A dose-dependent reduction in phospho-T398-S6K levels was observed and the degree of inhibition increased with longer treatment time. For all western blots, relative band intensity was estimated using Image J. (B) Western blot analysis of phospho-S6K in different body parts of *w^Dah^* flies. Flies were maintained with or without 200 μM rapamycin for 2 weeks prior to preparation of protein extracts from heads, thoraces, and abdominal segments. Rapamycin was found to efficiently reduce levels of phospho-T398-S6K in all fly body parts.

We measured phosphorylation of S6K, a well-described downstream target of TORC1, as an indicator of rapamycin efficacy, by western blot analysis using a phospho-Thr398-dependent S6K antibody. We observed a significant dose-dependent reduction in phospho-T398-S6K levels after rapamycin treatment for 1–3 days, confirming that rapamycin reduced TOR signaling in vivo (Figure 1A). To determine bioavailability of rapamycin throughout the fly, we measured S6K phosphorylation in different regions of the fly body. Phospho-T398-S6K levels were decreased to similar levels in heads, thoraces, and abdomens (Figure 1B), suggesting that TOR signaling is ubiquitously downregulated in adult flies upon rapamycin treatment.

### Rapamycin Extends Life Span, Increases Stress Resistance, Reduces Fecundity, and Increases Lipid Levels

**Figure 2:**
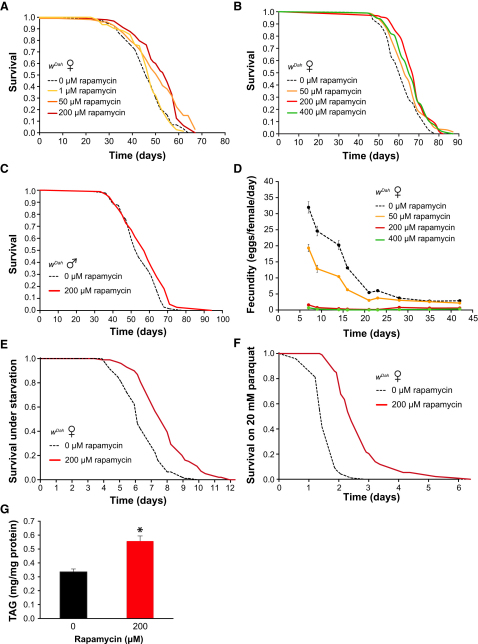
Effects of Rapamycin on Life Span and Fecundity in Various Standard Laboratory Strains (A) Rapamycin treatment extends the life span of *w^Dah^* females. Compared to flies on control food (0 μM rapamycin), flies on 50 and 200 μM rapamycin food have increased median life spans (p = 0.0005 and p < 0.0001, log-rank test). (B) Rapamycin treatment at 50, 200, and 400 μM extends the life span of *w^Dah^* females (p = 0.0428, p < 0.0001, p = 0.0013, log-rank test compared to control). (C) Rapamycin extends life span in *w^Dah^* males (p = 0.0241, log-rank test). (D) Reduced fecundity of females on 50, 200, and 400 μM rapamycin food. Data are given as mean number of eggs laid per female per day ± SEM. T test p values between egg laying under different conditions are p < 0.005. For each time point and each condition, eggs from ten vials containing ten flies per vial were counted. (E) Rapamycin enhances starvation resistance (p < 0.0001, log-rank test). For all stress assays, *w^Dah^* females were pretreated for 2 weeks with 200 μM rapamycin, and initial population was 100 flies per condition. (F) Rapamycin pretreated *w^Dah^* females have improved survival on paraquat (p < 0.0001, log-rank test). (G) Rapamycin treatment increases TAG levels. TAG levels in *w^Dah^* flies were measured after 14 days of rapamycin treatment from heads and thoraces of five females. Twelve samples were measured per treatment. Data are shown as mean ± SEM. ^∗^ indicates statistical significance of difference between untreated and rapamycin-treated flies (t test, p < 0.05).

We next investigated the effects of rapamycin on life span when administered at different concentrations. Whereas 1 μM rapamycin had no effect, significant life span extension occurred at 50, 200, and 400 μM in repeated experiments (Figures 2A and 2B), with no significant differences in survival between flies exposed to the three effective concentrations. In subsequent experiments, we used 200 μM rapamycin, which produced the largest increase in median life span. To test the generality of the effect of rapamycin on life span, we examined two other, frequently used laboratory strains, *w^1118^* and *yw*, and observed significant increases in median life span in both (Figures S2A and S2B). Rapamycin also increased life span of male flies, less so than that of females (Figure 2C). This sexual dimorphism is also observed in mice, where rapamycin-mediated life span extension is smaller in males than in females (Harrison et al., 2009), and only female S6K1 mutant mice are long lived (Selman et al., 2009). Furthermore, we found that removal of the cytoplasmic endosymbiont *Wolbachia*, which can modulate life span of some *Drosophila* mutants (Ikeya et al., 2009; Toivonen et al., 2007), did not affect extension of life span by rapamycin (Figures S2C and S2D). Thus, rapamycin consistently extended fly life span in diverse genetic and cytoplasmic backgrounds and in both sexes.

Rapamycin also reduced female fecundity in a dose-dependent manner, with a mild decrease at 50 μM and much more severe effects at 200 and 400 μM (Figure 2D and Figure S2F). However, since the three concentrations of rapamycin had similar effects on life span, there was no simple correlation between fecundity and longevity, arguing against a causal connection between them. Moreover, rapamycin extended the life span of sterile females carrying the *ovo^D^* mutation (Figure S2E), further confirming that extension of life span by rapamycin cannot be simply explained by reduced fecundity.

Interventions that extend fly life span are often associated with resistance to various stresses (Broughton et al., 2005; Clancy et al., 2001). We therefore tested rapamycin-treated flies for survival under starvation and treatment with paraquat, an oxidative stress inducer. Flies were pretreated with rapamycin for 2 weeks and then transferred to either agar-only food for starvation assays or to food supplemented with 20 mM paraquat. Pretreatment with rapamycin significantly increased survival under both starvation and paraquat treatment (Figures 2E and 2F). Furthermore, pretreatment with rapamycin increased survival after paraquat injection, ruling out any effect of rapamycin on feeding behavior as an explanation (Figure S2G).

The TOR pathway is a key controller of cellular metabolism, by coordinating nutrient supply and available energy with cellular demands for protein synthesis and growth. Consequently, it affects fat metabolism and energy reserves of triglycerides, which may enable animals to survive for longer in periods of starvation (Wullschleger et al., 2006). We therefore measured triacylglyceride (TAG) levels in flies treated with rapamycin and, in agreement with others (Teleman et al., 2005), we found that rapamycin treatment resulted in significantly elevated TAG levels (Figure 2G).

### Rapamycin Treatment Mediates Life Span Extension Independently of Effects on IIS and AMPK

**Figure 3:**
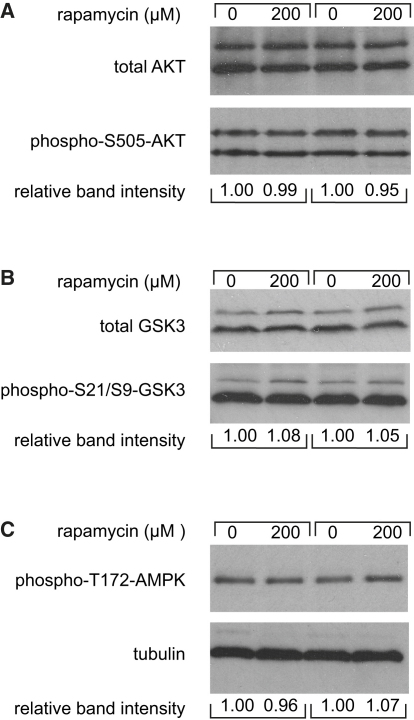
Rapamycin Treatment Does Not Affect IIS Signaling or AMPK Activity Western blot analyses of downstream components of IIS signaling and of AMPK phosphorylation. Protein extracts were made from whole flies after 14 days of rapamycin treatment. No changes in the levels of phospho-Ser505-Akt (A), phospho-Ser21/S9-GSK3 (B), or phospho-T172-AMPK (C) were observed. The presence of two bands for AKT is due to two isoforms present in flies (A). GSK3 has several isoforms in flies, and two of them are detected in this blot (B).

Rapamycin is generally thought to be a specific inhibitor of TORC1, whereas TORC2 is considered to be rapamycin insensitive (Wullschleger et al., 2006). However, in some cell lines, prolonged rapamycin treatment can also inhibit TORC2 activity (Sarbassov et al., 2006). Because we administered rapamycin from early adulthood until death, and also because TORC2 has been implicated in aging in worms (Soukas et al., 2009), we determined whether TORC2 activity was reduced by rapamycin. TORC2 phosphorylates and activates AKT kinase at Ser505. Western blot analysis of whole-fly protein extracts did not reveal a measurable effect of rapamycin on TORC2-specific phosphorylation of Akt at Ser505 (Figure 3A). We found no effect of rapamycin on phosphorylation of GSK3α/β at the Ser21/9 site, an output of IIS/Akt activity (Figure 3B). Taken together, these data suggest that neither TORC2 nor IIS was affected by rapamycin treatment.

AMPK is a cellular energy sensor that is activated when the AMP/ATP ratio is high. Subsequently, activated AMPK inhibits TOR signaling by activating phosphorylation of the TOR suppressor TSC2, thereby adjusting the cell's metabolic program to energy status (Towler and Hardie, 2007). Conversely, S6K regulates AMPK by changing the AMP/ATP ratio (Aguilar et al., 2007). Since S6K activity was downregulated upon rapamycin administration, we examined the phosphorylation status of the catalytic subunit of AMPK at the Thr172 site, to determine if rapamycin treatment activated AMPK. We observed no changes in AMPK phosphorylation after prolonged rapamycin treatment (Figure 3C), excluding AMPK activation as a possible effector of rapamycin-mediated effects on longevity.

### Extension of Life Span by Rapamycin Is Mediated by Several Downstream Targets of TORC1

**Figure 4:**
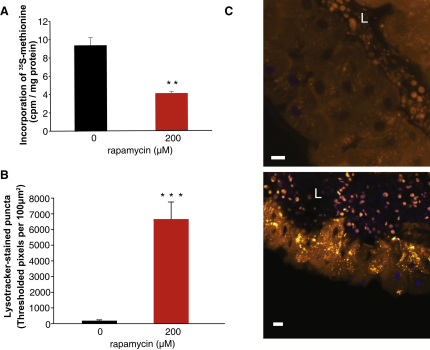
Rapamycin Increases Autophagy and Decreases Protein Translation (A) Levels of ^35^S-methionine incorporation in 7-day-old flies normalized to total protein content (±SEM). Rapamycin significantly reduces ^35^S-methionine incorporation (^∗∗^p = 0.0033, Student's t test). (B) Significantly more autolysosomes and lysosomes in rapamycin-treated flies (^∗∗∗^p < 0.0001, Student's t test). Average number of LysoTracker-stained puncta in fly midguts isolated from rapamycin pretreated or control flies is presented (±SEM). (C) Representative confocal fluorescence images of fly midgut stained with LysoTracker Red. Midgut of control flies (upper image) and rapamycin pretreated flies (lower image) are shown. L indicates lumen of the gut; scale bar is 10 μM.

Inhibition of TOR signaling has pleiotropic effects on cell physiology, including activation of autophagy (Klionsky, 2007), decreased cap-dependent translation, and increased cap-independent translation (Sonenberg and Hinnebusch, 2009). These changes could all potentially contribute to rapamycin-mediated life span extension. We first examined the effects of rapamycin on translation and autophagy. Rapamycin treatment produced a significant decrease in the incorporation of ^35^S-methionine, suggesting that rapamycin treatment inhibited translation (Figure 4A). There was also a significant increase in the number of LysoTracker-stained lysosomes and autolysosomes after rapamycin treatment, indicative of autophagy induction (Figures 4B and 4C). Rapamycin treatment thus affected physiological processes downstream of TORC1.

**Figure 5:**
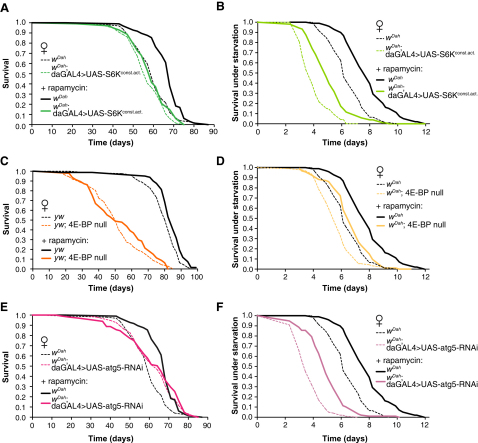
Rapamycin-Mediated Life Span Extension Is Blocked by the Ubiquitous Overexpression of Constitutively Active S6K, the Absence of 4E-BP, or Downregulation of *Atg5* (A) Ubiquitous overexpression of a constitutively active form of S6K (*UAS-S6K^const.act.^*) by the *daughterless*-GAL4 (*da*GAL4) driver abolishes rapamycin-mediated life span extension. Rapamycin increases median life span of *w^Dah^* females (p < 0.0001, log-rank test), but not *da*GAL4 > *UAS-S6K^const.act^*· (p = 0.1083, log-rank test). On control 0 μM rapamycin food, overexpression of constitutively active S6K slightly decreases life span (p = 0.0463, log-rank test). (B) Flies overexpressing constitutively active S6K are more sensitive to starvation compared to *w^Dah^* control flies (p < 0.0001, log-rank test), and their starvation resistance is improved by rapamycin (p < 0.0001, log-rank test). (C) Rapamycin does not extend the life span of 4E-BP null mutant female flies (p = 0.4027, log-rank test) but does increase the life span of control *yw* flies (p = 0.0033, log-rank test). (D) 4E-BP null flies are sensitive to starvation compared to control flies (p < 0.0001, log-rank test), but rapamycin improves their starvation resistance (p < 0.0001, log-rank test). (E) Downregulation of autophagy abolishes rapamycin-mediated life span extension (p = 0.5383, log-rank test). Autophagy was downregulated by ubiquitous overexpression of UAS-*atg5*-RNAi using *daughterless*-GAL4 (*da*GAL4 > UAS-*atg5*RNAi). Note that since the experiments were run in parallel, life span data for wild-type controls are the same as in Figure 4A. (F) Flies with ubiquitous overexpression of UAS-*atg5*-RNAi (*da*GAL4 > UAS-*atg5*RNAi) were more starvation sensitive compared to their controls (p < 0.0001, log-rank test), and their starvation resistance was improved by rapamycin treatment (p < 0.0001, log-rank test). Note that these experiments were run in parallel to those in Figures 4B and 4D, hence the life span data for controls are the same.

We next determined the processes that mediated life span extension by rapamycin. S6K mediates the downstream effects of TOR signaling on translation initiation, and downregulation of protein translation has been shown to increase life span in worms (Hansen et al., 2007; Kaeberlein and Kennedy, 2008; Syntichaki et al., 2007). We therefore examined the effects of rapamycin on flies ubiquitously overexpressing a constitutively active form of S6K (Barcelo and Stewart, 2002), and found that rapamycin had no significant effects on life span (Figure 5A), suggesting that rapamycin extended life span by downregulation of S6K activity. Downregulation of TOR signaling leads to 4E-BP activation, resulting in inhibition of cap-dependent translation (Sonenberg and Hinnebusch, 2009). We therefore administered rapamycin to 4E-BP null mutant flies (Tettweiler et al., 2005) and found that it did not significantly extend their life span (Figure 5C), suggesting that 4E-BP also mediates extension of life span by rapamycin.

Rapamycin has been shown to upregulate autophagy, an evolutionarily conserved cellular process that removes damaged proteins and entire organelles from cells and that may play a role in life span extension (Diaz-Troya et al., 2008; Ravikumar et al., 2004; Simonsen et al., 2008; Toth et al., 2008). We therefore determined whether upregulation of autophagy mediated extension of life span by rapamycin. ATG5 is required for formation of autophagosomes and is specific and essential for autophagy (Reggiori and Klionsky, 2005). Ubiquitous double-stranded RNA interference against *Atg5*, which inhibits autophagy induction (Ren et al., 2009; Scott et al., 2004), resulted in an approximate 2-fold reduction in *Atg5* expression as determined by QPCR (Figure S3) and completely abrogated the extension of life span by rapamycin (Figure 5E), suggesting that upregulation of autophagy mediates the life span extension.

Interestingly, all of the genetic models in which we manipulated the activity of downstream targets of TORC1 were starvation sensitive; flies overexpressing a constitutively active S6K, 4E-BP nulls, and flies with reduced expression of *Atg5* were more sensitive to starvation than their controls (Figures 5B, 5D, and 5F). Although rapamycin treatment did not increase life span in these flies, it did significantly increase their starvation resistance (Figures 5B, 5D, and 5F), demonstrating that these two traits can be uncoupled.

### Rapamycin Treatment Can Further Extend Life Span of Some Long-Lived IIS Mutants

**Figure 6:**
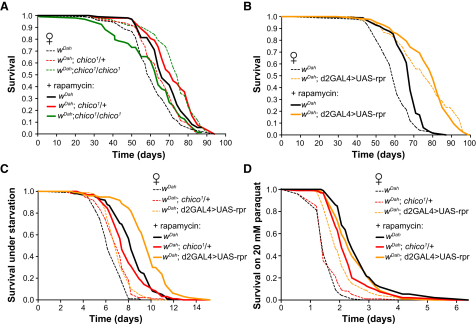
Effect of Rapamycin Treatment on Long-Lived IIS Mutants (A) Rapamycin extends the life span of long-lived *chico^1^* heterozygotes, (p = 0.0005) but decreases the life span of *chico^1^/chico^1^* homozygotes (p < 0.0001). On control food, *chico^1^* heterozygotes (p = 0.039) and *chico^1^*/*chico^1^* nulls (p < 0.0001) have increased median life span compared to control *w^Dah^* flies. Log-rank test is used for analyses. (B) Rapamycin does not extend the life span of mNSC-ablated flies. mNSCs were partially ablated by driving expression of the proapoptotic factor *reaper* (UAS-*rpr*) in the mNSCs using the *dilp2*-GAL4 driver (*d2*GAL4). On control food, mNSC-ablated flies are long lived (p < 0.0001, log-rank test compared to control). Log-rank test p values for rapamycin-treated versus nontreated flies of the same genotype are p < 0.0001 for *w^Dah^* and p = 0.97 for *d2*GAL4 > UAS-*rpr*. (C) Flies pretreated with 200 μM rapamycin survived longer under starvation conditions compared to untreated flies. Log-rank statistics for nontreated versus rapamycin-pretreated flies for all genotypes are p < 0.0001. Compared to wild-type controls, both *chico^1^* heterozygotes and mNSC-ablated flies were more resistant to starvation without rapamycin pretreatment (p = 0.006 and p = 0.001, respectively, log-rank tests). (D) Flies pretreated with 200 μM rapamycin survive longer when fed 20 mM paraquat. Log-rank statistics for nontreated versus rapamycin pretreated flies for all genotypes are p < 0.0001. Compared to wild-type controls, mNSC-ablated flies were more resistant to paraquat ingestion without rapamycin pretreatment (p < 0.0001, log-rank test).

Downregulation of IIS can extend life span in diverse organisms (Piper et al., 2008). Because of the many potential interactions between the TOR pathway and IIS, we investigated whether rapamycin treatment could further increase the life span of long-lived IIS mutants. Rapamycin treatment of flies heterozygous for *chico^1^*, a null mutation in the gene encoding the single *Drosophila* IRS homolog, extended life span beyond that induced by rapamycin treatment of controls (Figure 6A). However, rapamycin treatment of *chico^1^* homozygotes, which have more strongly reduced IIS and longer life span than heterozygotes (Figure 6A), shortened life span to levels comparable with non-rapamycin-treated controls (Figure 6A). Rapamycin treatment was biochemically effective in *chico^1^* homozygotes, because phosphorylation of S6K was similarly downregulated by rapamycin (Figure S4). Rapamycin treatment of flies with reduced levels of *Drosophila* insulin-like peptides (DILPs) due to the partial ablation of the DILP-producing median neurosecretory cells (mNSC) (Broughton et al., 2005) did not further increase life span in these long-lived flies (Figure 6B). Taken together, these data suggest that, when life span by rapamycin treatment is maximized, weak downregulation of IIS, as in *chico^1^* heterozygotes, can further extend life span, showing that IIS extends life span by mechanisms additional to those affected by rapamycin. However, stronger downregulation of IIS, which alone extends life span, can be deleterious in the presence of rapamycin, through as-yet-unidentified mechanisms.

We also examined the effects of rapamycin treatment on stress resistance in IIS mutants. Rapamycin treatment increased survival under starvation (Figure 6C) and paraquat treatment (Figure 6D) not only of *chico^1^* heterozygotes but also mNSC-ablated flies, despite not causing an effect on life span of the mNSC-ablated flies. The effects of these two interventions on life span can thus be uncoupled from their effects on resistance to starvation and paraquat treatment.

### Rapamycin Extends Life Span beyond the Maximum Achieved by Dietary Restriction

**Figure 7:**
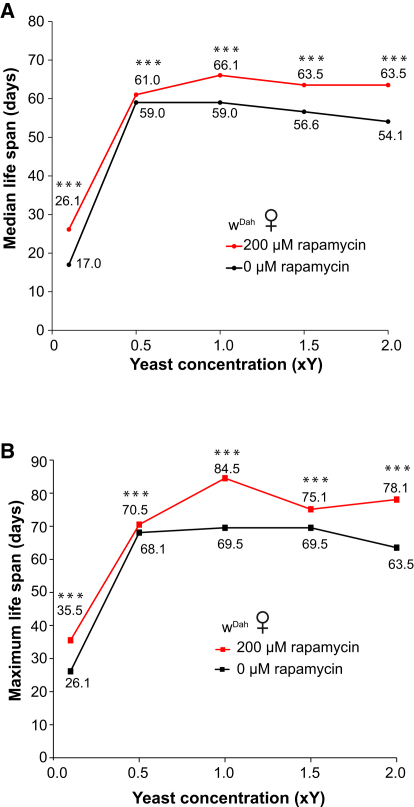
Rapamycin Increases Life Span Irrespective of Food Concentration Rapamycin increases (A) median and (B) maximum life span across different yeast concentrations in SYA food. Plotted are median life span values for *w^Dah^* against yeast concentration (0.1×, 0.5×, 1.0×, 1.5×, and 2.0× yeast) in SYA food (black line) and on the same food concentrations but supplemented with 200 μM rapamycin (red line). Values on each of the curves represent median (A) or maximum (B) life span. Flies on 200 μM rapamycin food had significantly increased life span at each yeast concentration (^∗∗∗^p < 0.001, log-rank test). For detailed statistical analyses and complete survival curves, please see Figure S5. Maximum life span is defined as the median of the last 10% survivorship.

The TOR pathway may play an important role in DR, because the effects of DR are abrogated in TOR pathway mutant yeast, worms, and flies (Bishop and Guarente, 2007; Mair and Dillin, 2008). We therefore examined whether DR affected the life span of rapamycin-treated flies (Figure 7 and Figure S5). Rapamycin significantly extended median and maximum life span at all food concentrations tested (Figure 7), although the extension was lowest at the food concentration that maximized life span of untreated controls. The highest food concentration (2.0xY) shortened the life span of rapamycin-treated flies by only 4% as opposed to 8% in controls, a highly significant difference. Thus, rapamycin slightly extended life span of flies that had already been maximized by DR and maintained life span in the presence of increased food.

## Discussion

The most well-validated antiaging interventions involve two major nutrient-sensing pathways: IIS and TOR. Although strong alterations of either of these signaling pathways can cause adverse effects, including embryonic lethality, cancer, and diabetes, milder downregulation can be beneficial for health and longevity in all organisms so far tested (Piper et al., 2008). An important consequence of using IIS or TOR manipulations as antiaging interventions is that they can also delay the progression of numerous diseases associated with old age, for example, cancer, neurodegeneration, cardiovascular disease, and diabetes (Bishop and Guarente, 2007; Piper et al., 2008). Hence, clarifying the role of the TOR pathway in aging, as well as its relationship with IIS, could provide valuable insights for developing treatments for age-related diseases.

The aim of this study was to determine if a pharmacological intervention to reduce TOR signaling had antiaging effects in *Drosophila* and to identify the underlying mechanisms. Rapamycin was chosen because it is the most specific and best-studied TOR kinase inhibitor available (Hartford and Ratain, 2007; Wullschleger et al., 2006). Importantly, rapamycin analogs have already been approved for human use as immunosuppressant drugs and are currently under clinical trials for use as anticancer agents (Guertin and Sabatini, 2009).

We administered rapamycin to adult *Drosophila* and demonstrated a dose-dependent downregulation of TOR activity after just 1 day of treatment by measuring the phosphorylation status of the TORC1 target, S6K. TOR inhibition by rapamycin was ubiquitous, since the levels of phosphorylated S6K were similarly downregulated in all main segments of the fly body. Notably, we found that continuous rapamycin treatment from early adulthood resulted in a robust and reproducible extension of life span, independent of the genetic and cytoplasmic background and sex of the flies. Rapamycin treatment in yeast has been previously shown to extend replicative (Medvedik et al., 2007) and chronological (Powers et al., 2006) life span, and a recent study has shown that rapamycin also extends life span in mice when administered late in life (Harrison et al., 2009). These data, together with our observation that rapamycin treatment also extends life span in *Drosophila*, suggest that the antiaging effects of rapamycin are evolutionarily conserved.

Rapamycin treatment that increased life span also reduced female fecundity. However, rapamycin also extended life span of sterile *ovo^D^* females, strongly suggesting that reduced fertility alone is not sufficient for rapamycin-mediated life span extension. In *C. elegans* also, downregulation of TOR, ribosomal proteins, S6K, or elF genes decreases fecundity, and reduced TOR signaling can extend life span in sterile mutants (Hansen et al., 2007). In addition, a growing body of evidence suggests that reduced fecundity and longevity can be uncoupled (Grandison et al., 2009; Partridge et al., 2005).

TOR kinase is a central component of two protein complexes: TORC1 and TORC2. Rapamycin is generally considered to be a specific inhibitor of TORC1, although in some cell lines prolonged rapamycin treatment can also inhibit TORC2 activity (Sarbassov et al., 2006). Rapamycin treatment significantly reduced S6K phosphorylation, indicative of reduced TORC1 activity. However, we saw no change in phosphorylation of Akt at a TORC2-specific phosphorylation site, suggesting that TORC2 activity remained unchanged. Both TORC1 and TORC2 interact with components of the IIS pathway: the TORC2 complex phosphorylates and activates Akt kinase; conversely, Akt phosphorylates and functionally inactivates the TOR pathway suppressor protein TSC2. In addition, S6K inhibits IRS at the level of translation, transcription, and phosphorylation, thereby exerting a negative feedback loop on IIS (Um et al., 2006; Wullschleger et al., 2006). Reduced IIS could therefore contribute to extension of life span by rapamycin. However, we did not detect any measurable change in GSK3α/β phosphorylation, an important downstream target of the IIS pathway, suggesting that rapamycin treatment did not alter IIS output.

We performed epistasis experiments to explore the functional interactions between TOR and IIS in determination of life span. When life span extension was maximized by rapamycin treatment, the effects of IIS manipulation were dependent upon the degree of IIS downregulation. Thus, while rapamycin treatment further extended the life span of flies heterozygous for *chico^1^*, a null mutation in the gene encoding the single *Drosophila* IRS homolog, it shortened the life span of long-lived *chico^1^* homozygous flies. Rapamycin treatment was hence beneficial under conditions of weaker IIS downregulation, possibly indicating deleterious combined effect with stronger IIS downregulation. Alternatively, extension of life span by rapamycin may require *chico^1^*. Rapamycin did not alter the life span of flies in which the insulin-producing mNSCs were partially ablated, possibly because they are intermediate in degree of downregulation of IIS. Since life span under rapamycin treatment was maximized, the data from *chico^1^* heterozygotes suggest that IIS and TOR regulate life span, at least in part, by nonoverlapping mechanisms. In *C. elegans,* life span extension of *daf-2* (the worm insulin receptor) mutants was not modified by RNAi against TOR, suggesting that these two pathways may have overlapping downstream targets (Hansen et al., 2007; Vellai et al., 2003). The IIS and TOR pathways may converge on a common downstream target, with severe inhibition of the two pathways causing detrimental effects, for example, through the induction of apoptosis (Talapatra and Thompson, 2001). An important downstream effector of IIS-mediated life span extension is the Forkhead transcription factor Foxo/DAF-16. However, in *C. elegans*, the life span of *daf-16* null mutants can be extended by reductions in TOR signaling (Hansen et al., 2007), and, similarly, rapamycin treatment can extend the life span of dFOXO null flies (C.S. and L.P., unpublished data), suggesting that Foxo is not required for rapamycin to extend life span.

Inhibition of TOR signaling via TORC1 has pleiotropic effects on cell physiology, including activation of autophagy (Klionsky, 2007), downregulation of cap-dependent protein translation, and increased cap-independent translation (Sonenberg and Hinnebusch, 2009). All of these changes could potentially contribute to the antiaging effects of rapamycin (Hansen et al., 2007, 2008; Kaeberlein and Kennedy, 2008; Kapahi et al., 2004).

Elevated levels of autophagy are generally considered to be beneficial for the prevention of aging, due to increased rates of removal of damaged molecules and organelles (Klionsky, 2007). For example, upregulation of *Atg8* in fly neurons extends life span and is associated with decreased amounts of insoluble ubiquinated and oxidatively damaged proteins (Simonsen et al., 2008). Furthermore, downregulation of autophagy in *C. elegans* shortens the life span of long-lived *daf-2* mutant worms (Hansen et al., 2008; Melendez et al., 2003), suggesting that autophagy is required for the longevity effects of IIS mutants. However, higher levels of autophagy alone do not appear to be sufficient for increased life span, because *daf-16* mutation blocks *daf-2* longevity but does not reduce autophagy levels (Hansen et al., 2008). Rapamycin induces autophagy, possibly by altering the interaction of TORC1 with the autophagy proteins ATG13 and ATG1 (Chang and Neufeld, 2009; Ravikumar et al., 2004). We have shown that downregulation of autophagy blocks rapamycin-mediated life span extension. Similar observations have also been made for chronological life span in yeast (Alvers et al., 2009). It is interesting to note that, although autophagy seems to be an important contributor to longevity (Toth et al., 2008), we did not observe shortening of life span upon reduced expression of *Atg5*, which is in agreement with the previously published observations (Ren et al., 2009).

Reduced protein translation can also have an antiaging effect. Mutations in genes encoding ribosomal proteins, S6K, or translation initiation factors, which are involved in translation, can all extend life span, in yeast, worms, and flies (Kaeberlein and Kennedy, 2008). However, the precise mechanisms underlying the antiaging effects of reduced translation remain elusive, although several possible mechanisms have been proposed. For example, energy saved by lowering translation may be reinvested in longevity-promoting processes; stresses imposed by mutations in the translation machinery may result in cap-independent translation and enrichment of different sets of proteins; and improved protein homeostasis and better removal of damage may allow for increased life span when translation is altered (Kaeberlein and Kennedy, 2008). Rapamycin has been shown to be a potent repressor of translation; both microarray and proteomic analyses have demonstrated that rapamycin significantly decreases translation of mRNA-encoding initiation factors and ribosomal proteins (Grolleau et al., 2002; Guertin et al., 2006). This effect on translation is mediated by the downstream targets of TORC1: S6K and 4E-BP (Fingar and Blenis, 2004; Wullschleger et al., 2006). We have shown that both a constitutive upregulation of S6K activity and the absence of 4E-BP block rapamycin-mediated life span extension, suggesting that downregulation of protein translation is an important mediator of the effects of rapamycin on life span.

Thus, we have demonstrated that the positive in vivo effects of rapamycin on life span are mediated by the TORC1-dependent downstream processes, autophagy and protein translation. However, it is not yet clear if the effects of autophagy and protein translation are additive or if their combined effects are required to extend life span. Downregulation of S6K alone is sufficient to extend life span in flies (Kapahi et al., 2004). However, S6K activity modulates the activity of ATG1 kinase, an important regulator of autophagy, which may thus be affected in addition to translation (Lee et al., 2007). Thus, these processes may act in concert to extend life span: autophagy may reduce cellular damage and thereby provide cells with ATP and amino acids, which can subsequently be used for cap-independent translation, stimulating the synthesis of proteins that are important for stress resistance and, perhaps, longevity.

Interestingly, rapamycin treatment increased median and maximum life span under DR. Life span of rapamycin-treated flies was increased at all food concentrations, suggesting that the effect of rapamycin on life span is at least partially independent of the effects of DR. Therefore, rapamycin treatment appears to capture all of the advantages of DR plus additional benefits. Moreover, although rapamycin-treated flies still responded to DR, the DR response was less pronounced than in controls, and, in particular, the rapamycin-treated flies were more resistant to the effects of full feeding on mortality. The mechanisms underlying DR and its interactions with the TOR pathway are complex. For example, DR in *C. elegans* cannot extend the life span of long-lived TOR RNAi worms, but it does nevertheless extend the life span of mutants that have reduced levels of S6K and ribosomal proteins (Bishop and Guarente, 2007; Hansen et al., 2007; Mair and Dillin, 2008). In *Drosophila*, life span extension by DR cannot be increased further by ubiquitous overexpression of the TOR suppressor, TSC2 (Kapahi et al., 2004). However, our data demonstrate that rapamycin treatment may involve additional longevity assurance pathways, because it can increase life span beyond the maximum achieved by DR. Interestingly, it was recently shown that 4E-BP extends life span under DR by enhancing mitochondrial activity (Zid et al., 2009), which may underlie part of the beneficial effects of rapamycin.

In conclusion, we have shown that rapamycin treatment delays aging, an effect more pronounced than DR and that could further increase life span in combination with mild downregulation of IIS, firmly demonstrating that rapamycin could be used to study aging in flies. Future studies to investigate the effects of rapamycin in disease model systems may reveal potential common therapies for a wide range of age-related conditions.

## Experimental Procedures

### Fly Stocks and Husbandry

The wild-type stock *Dahomey* was collected in 1970 in Dahomey (now Benin) and has since been maintained in large population cages with overlapping generations on a 12L:12D cycle at 25°C. The *white* Dahomey (*w^Dah^*) stock was derived by incorporation of the *w^1118^* mutation into the outbred Dahomey background by backcrossing. All stocks were maintained and all experiments were conducted at 25°C on a 12 hr:12 hr light:dark cycle at constant humidity using standard sugar/yeast/agar (SYA) media (Bass et al., 2007). For all experiments, flies were reared at standard larval density, and eclosing adults were collected over a 12 hr period. Flies were mated for 48 hr before sorting into single sexes. Further details concerning fly mutants used can be found in the Supplemental Experimental Procedures.

### Rapamycin Treatment

Rapamycin (LC Laboratories) was dissolved in ethanol and added to SYA food at appropriate concentrations (1, 50, 200, or 400 μM). For control food (0 μM), ethanol alone was added.

### Stress Assays

For stress assays, flies were reared and housed as for life span experiments. Flies were pretreated with rapamycin at 200 μM for 14 days and then transferred to food supplemented with 20 mM methylviologen (paraquat, from Sigma) for oxidative stress assays, or to 1.5% agar for starvation assays.

### Dietary Restriction

The DR protocol was described in detail in Bass et al. (Bass et al., 2007).

### Statistical Analyses

Statistical analyses were performed using JMP software (version 4.0.5; SAS Institute). Log-rank tests were performed for survival. Other data were tested for normality using the Shapiro-Wilk W test on studentized residuals and, where appropriate, log transformed. One-way analyses of variance (ANOVA) and planned comparisons of means were made using Tukey-Kramer HSD test.

## Supplemental Information

Document S1. Five Figures, Two Tables, Supplemental Experimental Procedures, and Supplemental References
