# Supplementary material for: Mechanisms of Life Span Extension by Rapamycin in the Fruit Fly Drosophila melanogaster
Source: Cell Metab. 2010 Jan 6;11(1):35–46. doi: 10.1016/j.cmet.2009.11.010 (PMC2824086; doi:10.1016/j.cmet.2009.11.010)
Supplement: Document S1. Five Figures, Two Tables, Supplemental Experimental Procedures, and Supplemental References [file mmc1.pdf]

Cell Metabolism, Volume *11*

Supplemental Information

Mechanisms of Life Span Extension by Rapamycin in the Fruit Fly *Drosophila melanogaster*

Ivana Bjedov, Janne M. Toivonen, Fiona Kerr, Cathy Slack,
Jake Jacobson, Andrea Foley, and Linda Partridge

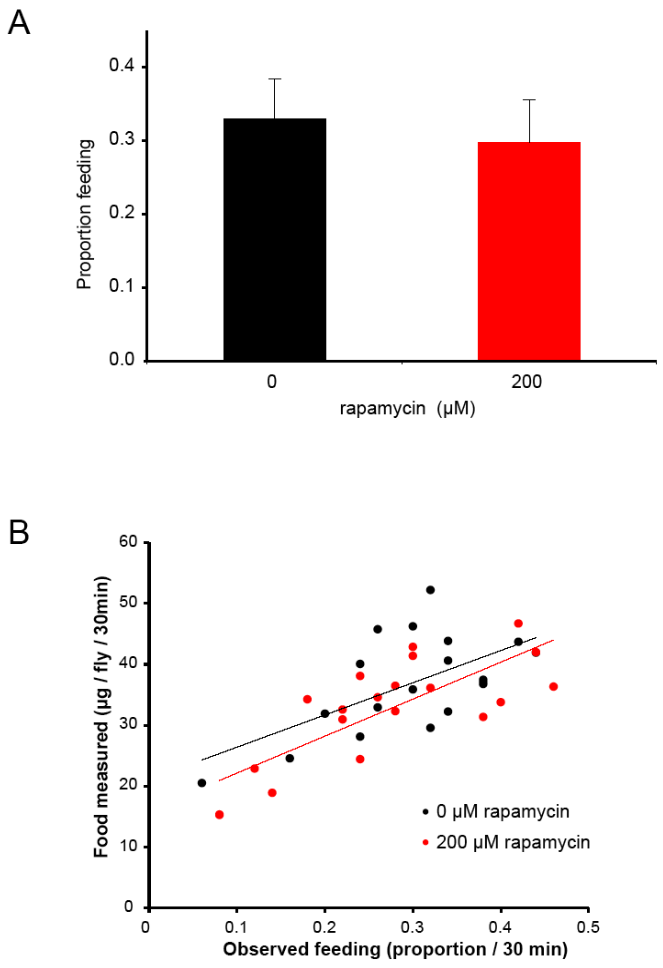

Bjedov et al., Figure S1

Figure S1. Feeding behaviour of w^{Dah} flies in the absence and presence of 200 µM rapamycin.

Flies were reared under standard density, as for lifespan experiments, and placed five per vial on standard food (see Materials and Methods). Feeding observations, using 7 day old females, were carried out on blue-dye-containing standard food that was supplemented or not with 200 µM rapamycin.

A) Figure represents feeding observations during a 30 minute period. Data are presented as proportion feeding (proportion of flies which were feeding / possible feeding events) ± SEM. There was no significant difference in the feeding behavior of flies on rapamycin food compared to flies on food not containing rapamycin ($p=0.95$, chi-square test).

B) Figure shows the relationship between the amount of food consumed by flies (as determined spectrophotometrically) and observed feeding behavior. There was a strong positive linear correlation between the volume of blue-dyed food consumed and the proportion of feeding events observed ($p<0.001$, linear model) that was unaffected by addition of 200 µM rapamycin to the food ($p=0.69$, linear model). There is no effect of interaction between the food type (standard food compared to standard food supplemented with 200 µM rapamycin) and the way blue dye accumulates in response to observed feeding events.

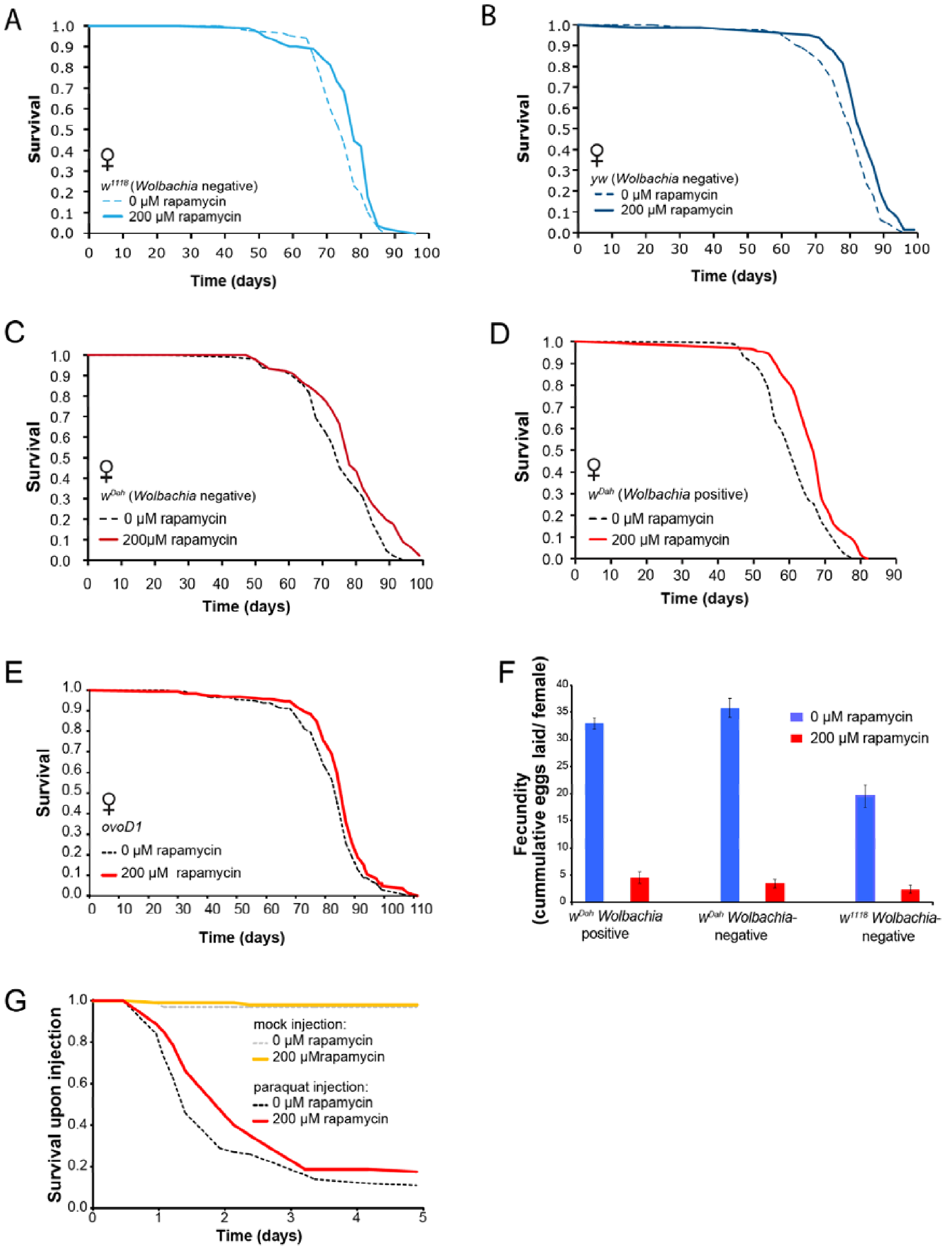

Bjedov et al., Figure S2

Figure S2. Rapamycin extends lifespan of different laboratory strains and of a sterile mutant, it reduces fecundity and improves survival upon paraquat injection.

A) Lifespan extension by rapamycin in laboratory strain *w*^{1118}. Median lifespans of *w*^{1118} females on control (0 μM rapamycin) food (dashed line) are 75 days (n=79) and on 200 μM rapamycin food (solid line) 78 days (n=86) (4% increase, p=0.0018, log-rank test).

B) Lifespan extension by rapamycin in laboratory strain *yw*. Median lifespans of *yw* females on control (0 μM rapamycin) food (dashed line) are 82 days (n=74) and on 200 μM rapamycin food (solid line) 85 days (n=74) (5% increase, p=0.0033, log-rank test).

C) and D) Lifespan extension by rapamycin was independent of *Wolbachia* status of the flies. Strains in the Figures C) and D) were genetically identical, except that *w*^{Dah} strain in C) received tetracycline treatment to remove endosymbiotic bacterium *Wolbachia*. When rapamcyin treated female flies were compared to non-treated controls, rapamycin was effective in extending lifespan of both *w*^{Dah} *Wolbachia* negative (p=0.0119, log-rank test) and *w*^{Dah} *Wolbachia* positive flies (p<0.0001, log-rank test). Lifespan in Figure D) is the same as in Figure 2B.

E) Rapamycin extends lifespan of long-lived sterile females carrying the *ovo*^{D} mutation (p=0.0085, log-rank test). The modest lifespan extension of *ovo*^{D} mutant flies by rapamycin is probably due to the different genetic background of the flies relative to the *w*^{Dah} controls and also because *ovo*^{D} females are inherently very long-lived mutants.

F) Rapamycin similary reduced egg-laying in different genetic backgrounds. *Wolbachia* status of *w*^{Dah} flies does not affect egg-laying. For this analysis, eggs from 5 vials from a 24-hour period were counted. Each contained 10 flies. Results represent cumulative egg-lay over three time points ± SEM.

G) Rapamycin increases survival after paraquat injection. Survivorship curves of 14-day old *w*^{Dah} female flies that were pretreated or not with 200 μM rapamycin and then injected with paraquat. Rapamycin pre-treatment significantly improved survival of flies after paraquat injections (p=0.03416, log-rank test). Number of flies tested was 60 for each mock control and 100 and 80 for rapamycin 0 μM and rapamycin 200 μM, respectively.

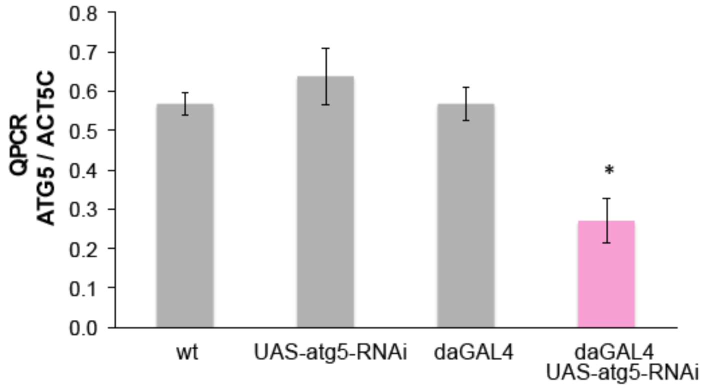

Bjedov et al., Figure S3

Figure S3. Efficacy of *atg5*-RNAi line.

Knockdown of *Atg5* by RNAi as measured by quantitative RT-PCR. *Atg5* transcript levels were measured in heads and thoraces of *da*GAL4>UAS-*atg5*RNAi flies (abdomens were not included because the RNAi construct is not expressed in the female germ-line) by quantitative RT-PCR. mRNA levels compared to *actin5C*. Data are shown as mean relative expression ± SEM, * denotes significant difference compared to controls ($p<0.05$). Expression level of *Atg5* in *da*GAL4>UAS-*atg5*RNAi flies was significantly lower than in all controls (one way ANOVA, Tukey's HSD test, $p<0.05$).

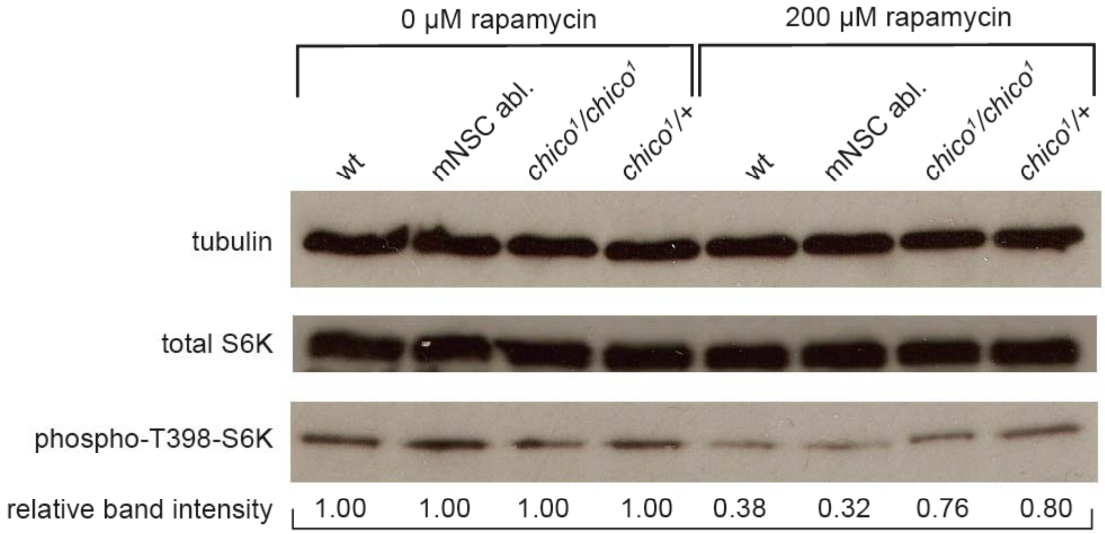

Bjedov et al., Figure S4

Figure S4. Reduction of S6K phosphorylation in IIS mutants upon rapamycin treatment.

Western blot analysis of phospho-T398-S6K, total S6K, and tubulin levels of whole fly protein extracts prepared from w^{Dah} control flies and IIS mutants ($chico^1$/+ heterozygotes, $chico^1/chico^1$ null, and mNSC-ablated flies). Flies were sampled after 18 days of 200 μM rapamycin treatment. For all western blots, relative band intensity was estimated using Image J.

A

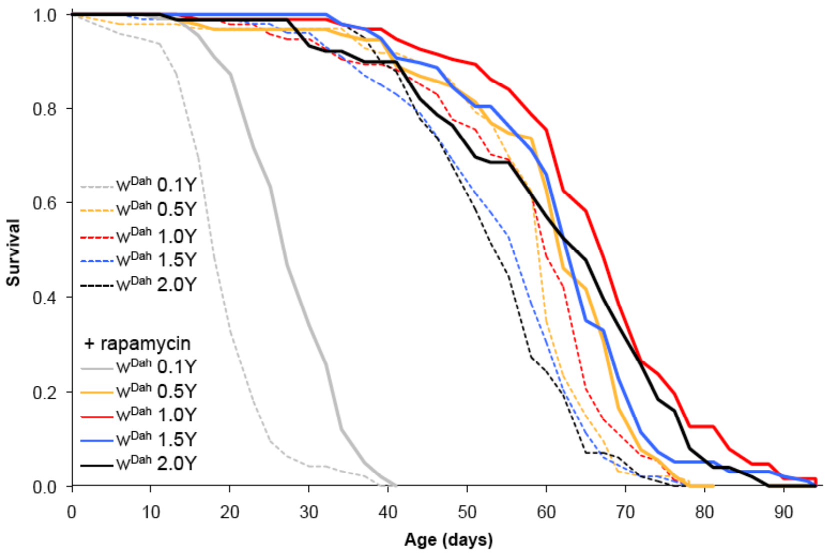

B

	w^{Dah} 0.5Y	w^{Dah} 1.0Y	w^{Dah} 1.5Y	w^{Dah} 2.0Y
w^{Dah} 0.1Y	9.71E-46	2.49E-45	9.43E-48	5.92E-51
w^{Dah} 0.5Y		0.2432	0.0339	0.0068
w^{Dah} 1.0Y			0.0031	0.0009
w^{Dah} 1.5Y				0.6507

	w^{Dah} rapamycin 0.5Y	w^{Dah} rapamycin 1.0Y	w^{Dah} rapamycin 1.5Y	w^{Dah} rapamycin 2.0Y
w^{Dah} rapamycin 0.1Y	9.35E-43	2.74E-47	4.94E-49	4.10E-40
w^{Dah} rapamycin 0.5Y		0.0001	0.4219	0.0320
w^{Dah} rapamycin 1.0Y			0.0081	0.0852
w^{Dah} rapamycin 1.5Y				0.3955

	w^{Dah} 0.1Y	w^{Dah} 0.5Y	w^{Dah} 1.0Y	w^{Dah} 1.5Y	w^{Dah} 2.0Y
w^{Dah} rapamycin 0.1Y	1.69E-16				
w^{Dah} rapamycin 0.5Y		0.0004			
w^{Dah} rapamycin 1.0Y			9.30E-09		
w^{Dah} rapamycin 1.5Y				1.30E-08	
w^{Dah} rapamycin 0.1Y					1.77E-08

C

	w^{Dah} 0.5Y	w^{Dah} 1.0Y	w^{Dah} 1.5Y	w^{Dah} 2.0Y
w^{Dah} 0.1Y	4.70E-14	9.10E-13	3.30E-13	6.80E-15
w^{Dah} 0.5Y		0.0239	0.8620	0.2580
w^{Dah} 1.0Y			0.0185	0.0013
w^{Dah} 1.5Y				0.1960

	w^{Dah}rapamycin 0.5Y	w^{Dah}rapamycin 1.0Y	w^{Dah}rapamycin 1.5Y	w^{Dah}rapamycin 2.0Y
w^{Dah} rapamycin 0.1Y	6.60E-12	2.27E-05	1.10E-14	5.28E-08
w^{Dah} rapamycin 0.5Y		5.74E-07	6.03E-08	1.86E-09
w^{Dah} rapamycin 1.0Y			0.0042	0.0053
w^{Dah} rapamycin 1.5Y				0.2870

	w^{Dah} 0.1Y	w^{Dah} 0.5Y	w^{Dah} 1.0Y	w^{Dah} 1.5Y	w^{Dah} 2.0Y
w^{Dah} rapamycin 0.1Y	4.61E-06				
w^{Dah} rapamycin 0.5Y		5.72E-04			
w^{Dah} rapamycin 1.0Y			6.11E-06		
w^{Dah} rapamycin 1.5Y				7.31E-09	
w^{Dah} rapamycin 0.1Y					1.23E-09

Bjedov et al., Figure S5

Figure S5. Complete survival curves and analysis of the effect of rapamycin lifespan extension of different food concentration.

A) Complete survival curves for the dietary restriction (DR) experiment that is presented in Figure 6. Plotted are survival curves for w^{Dah} females against yeast concentration (0.1x, 0.5x, 1.0x, 1.5x, and 2.0x yeast) in SYA food (dashed line) and on the same food concentrations but supplemented with 200 µM rapamycin (solid line).

B) Statistical analysis for median lifespan for DR experiment in Figure S5A and Figure 6. Significantly different comparisons for median lifespan ($p<0.05$, log-rank test) are highlighted in red. Rapamycin significantly extends median lifespan of laboratory strain w^{Dah} on all food concentration.

C) Statistical analysis for maximum lifespan for DR experiment in Figure S5A and Figure 6. Significantly different comparisons for maximum lifespan ($p<0.05$, log-rank test) are highlighted in red. Rapamycin significantly extends maximum lifespan of laboratory strain w^{Dah} on all food concentration.

Table S1. Statistical analysis of lifespan experiments for Figures 2, 4 and 5.

Figure	Genotype	Sex	Median	Number of flies	Rapamycin concentration in food (μM)	% increase over flies on control rapamycin 0μM food	Log-rank test comparison to control rapamycin 0μM food
**2A**	w^{Dah}	females	46	87	0		
	w^{Dah}	females	48	85	1	2%	p=0.86
	w^{Dah}	females	52	70	50	9%	p=0.0005
	w^{Dah}	females	55	63	200	13%	p<0.0001
**2B**	w^{Dah}	females	61	87	0		
	w^{Dah}	females	63	83	50	3%	p=0.0428
	w^{Dah}	females	67	76	200	10%	p<0.0001
	w^{Dah}	females	65	87	400	7%	p=0.0013
**2C**	w^{Dah}	males	54	80	0		
	w^{Dah}	males	57	84	200	6%	p=0.0241
**5A**	w^{Dah}	females	59	89	0		
	w^{Dah}	females	68	82	200	15%	p<0.0001
	w^{Dah}; daGAL4>UAS-S6K$^{const.act.}$	females	57	79	0		
	w^{Dah}; daGAL4>UAS-S6K$^{const.act.}$	females	59	78	200	4%	p=0.1083
**5C**	*yw*	females	82	74	0		
	yw	females	85	74	200	4%	p=0.0033
	yw; 4E-BP null	females	50	83	0		
	yw; 4E-BP null	females	50	88	200	0%	p=0.4027
**5E**	w^{Dah}; daGAL4>UAS-atg5-RNAi	females	66	84	0		
	w^{Dah}; daGAL4>UAS-atg5-RNAi	females	66	84	200	0%	p=0.5383
**6A**	w^{Dah}	females	60	74	0		
	w^{Dah}	females	66	74	200	10%	p=0.0005
	w^{Dah};chico^1/+	females	64	62	0		
	w^{Dah};chico^1/+	females	72	54	200	12.50%	p=0.0008
	w^{Dah};chico^1/*chico*^{1}	females	74	66	0		
	w^{Dah};chico^1/*chico*^{1}	females	64	82	200	-13.50%	p<0.0001
**6B**	w^{Dah}	females	59	89	0		
	w^{Dah}	females	68	82	200	15%	p<0.0001
	w^{Dah};d2GAL4>UAS-rpr	females	78	67	0		
	w^{Dah};d2GAL4>UAS-rpr	females	80	74	200	3%	p=0.97

Table S2. Statistical analysis of stress survival experiments for Figures 2, 4 and 5.

Figure	Genotype	Sex	Median	Number of flies	Food type *	Rapamycin pre-treatment	% increase over flies on control rapamycin 0 µM food	Log-rank test comparison to control rapamycin 0 µM food
**2E**	w^{Dah}	females	6	93	starvation assay			
	w^{Dah}	females	8	99	starvation assay	+	33%	p<0.0001
**2F**	w^{Dah}	females	1.4	96	paraquat assay			
	w^{Dah}	females	2.4	93	paraquat assay	+	70%	p<0.0001
**5B**	w^{Dah}	females	6	93	starvation assay			
	w^{Dah}	females	7.7	99	starvation assay	+	28%	p<0.0001
	w^{Dah}; daGAL4>UAS-S6K$^{const.act.}$	females	3.7	98	starvation assay			
	w^{Dah}; daGAL4>UAS-S6K$^{const.act.}$	females	4.6	104	starvation assay	+	24%	p<0.0001
**5D**	w^{Dah}; 4E-BP null	females	5.3	94	starvation assay			
	w^{Dah}; 4E-BP null	females	6.8	94	starvation assay	+	28%	p<0.0001
**5F**	w^{Dah}; daGAL4>UAS-atg5-RNAi	females	3.3	100	starvation assay			
	w^{Dah}; daGAL4>UAS-atg5-RNAi	females	4.6	99	starvation assay	+	39%	p<0.0001
**6C**	w^{Dah}	females	6	99	starvation assay			
	w^{Dah}	females	8.1	99	starvation assay	+	35%	p<0.0001
	w^{Dah};*chico*^{1}/+	females	6.8	99	starvation assay			
	w^{Dah};*chico*^{1}/+	females	7.3	97	starvation assay	+	7%	p<0.0001
	w^{Dah};d2GAL4>UAS-rpr	females	6.8	99	starvation assay			
	w^{Dah};d2GAL4>UAS-rpr	females	9.6	97	starvation assay	+	41%	p<0.0001
**6D**	w^{Dah}	females	1.4	96	paraquat assay			
	w^{Dah}	females	2.4	93	paraquat assay	+	70%	p<0.0001
	w^{Dah};*chico*^{1}/+	females	1.4	99	paraquat assay			
	w^{Dah};*chico*^{1}/+	females	2	95	paraquat assay	+	43%	p<0.0001
	w^{Dah};d2GAL4>UAS-rpr	females	1.6	101	paraquat assay			
	w^{Dah};d2GAL4>UAS-rpr	females	2.2	106	paraquat assay	+	38%	p<0.0001

* starvation assay: 1% (w/v) agar
paraquat assay: 20 mM paraquat in sugar-yeast-agar (SYA)

Bjedov et al. Table S2

Supplemental Experimental Procedures

Fly stocks and husbandry

w^{1118}, constitutively active S6K stocks (*w*^{1118}; P{UAS-S6k.TE}2 (#6912), *w*^{1118}; P{UAS-S6k.STDE}3 (#6913)), *daughterless*-GAL4 (*w*^{1118}; P{da-GAL4.w^{-}}3 (#8641)), and *ovo*^{D} strain (#1309) were obtained from the Bloomington *Drosophila* Stock Center (http://flystocks.bio.indiana.edu). The 4E-BP null mutant and its respective *yw* control were kindly provided by Paul F. Lasco (Tettweiler et al., 2005). The *Atg5*-RNAi strain (*UAS-Atg5*-IR) was provided by Thomas P. Neufeld (Ren et al., 2009; Scott et al., 2004). Other stocks used in this study (*chico*^{1}/*Cyo* (Bohni et al., 1999); UAS-*reaper (*UAS-*rpr)* and *dilp2*-GAL4 (Ikeya et al., 2002) were kind gifts from Ernst Hafen. Mutants and transgenes were backcrossed into *w*^{Dah} *Wolbachia* positive strain for at least eight generations. All stocks were maintained and all experiments were conducted at 25ºC on a 12h:12h light:dark cycle at constant humidity using standard sugar/yeast/agar (SYA) media (Bass et al., 2007). For all experiments, including lifespan experiments, flies were reared at standard larval density and eclosing adults were collected over a 12 hour period. Flies were mated for 48 hours before sorting into single sexes.

Wolbachia status of the flies was determined by PCR, as described in Toivonen et al. (Toivonen et al., 2007). While laboratory strains *w*^{1118} and *yw* were *Wolbachia* negative, *w*^{Dah} strain was *Wolbachia* positive. We have removed *Wolbachia* from *w*^{Dah} by tetracyclin treatment and at least five successive generations without tetracycline prior to lifespan experiments (Toivonen et al., 2007). As *Wolbachia* had no affect on rapamycin-mediated lifespan extention or egg-laying under our conditions, we have used *w*^{Dah} *Wolbachia* positive strains for our experiments.

Lifespan experiments

Flies were maintained in vials at a density of ten flies per vial. Flies were transferred to new vials every 2-3 days and scored for deaths. All lifespan experiments have been repeated at least twice, except *ovo*^{D} lifespan, *chico*^{1}/*chico*^{1} homozygotes lifespan and dietary restriction experiments.

Fecundity

Eggs were collected over 24-hour periods at several time-points during lifespan experiments. The number of eggs laid per vial at each time point was counted. For each condition and each time point, 5 (Figure S2F) or 10 (Figure 2D) vials were counted. Each vial contained 10 flies. Data are reported as the mean number of eggs laid per female fly per 24h ± SEM (Figure 2D) or as cumulative eggs laid per female fly (Figure S2F).

Lipid assays

For triacylglyceride (TAG) content quantification, batches of two flies, or heads and thoraces from 5-6 flies, were homogenized in 0.05% Tween according to Gronke et al. 2003. TAG content was quantified using the Triglyceride Infinity Reagent (ThermoScientific) using triglyceride standards and normalised to total protein content as determined using the BCA protein assay reagent (Pierce).

Western Blots

10 female flies were homogenized in 200μl of 2x Laemmli loading sample buffer (100 mM Tris 6.8, 20% glycerol, 4% SDS) containing 5% β-mercaptoethanol. Extracts were cleared by centrifugation and protein content determined by using the Bradford assay (Bio-Rad). Approximately 40µg of protein extract was loaded per lane on polyacrylamide gel. Proteins were separated and transferred to nitrocellulose membrane. Primary antibodies used were as follows: anti-AKT (Cell Signaling Technologies #9272, used at 1:1,000), anti-Ser505-phosphorylated *Drosophila* AKT (Cell Signaling Technologies #4054, 1:1,000), anti-phospho-Ser21/9-GSK-3-α/β (Cell Signaling Technologies #9331, 1:1,000), anti-GSK-3α/β (pan-Sgg/GSK3 4G1E11; gift of Marc Bourouis), anti-dS6K (gift of Thomas Neufeld 1:1,000), and anti-phospho-Thr398-S6K (Cell Signaling Technologies #9209, 1:1,000), anti-phospho-Thr172-AMPK (Cell Signaling Technologies #2353, 1:1,000), anti-tubulin (monoclonal mouse clone DM1A, Sigma, 1:2000). HRP-conjugated secondary antibodies (Abcam) were used. Blots were developed using the

ECL detection system (Amersham). Western blots were analysed using ImageJ program (US National Institutes of Health).

Quantitative RT-PCR

Live flies were snap frozen in liquid nitrogen and stored at -80°C . Total RNA from heads and thoraxes of 10 flies was extracted using TRIzol (GIBCO) according to the manufacturer's instructions. mRNA was reverse transcribed using oligo(dT) primer and the Superscript II system (Invitrogen). Quantitative PCR was performed using the Prism 7000 sequence-detection system (Applied Biosystems), SYBR Green (Molecular Probes), ROX ReferenceDye (Invitrogen), and Hot StarTaq (Qiagen, Valencia, CA) by following the manufacturers' instructions. Primers for *atg5* and *actin5C* used were as follows: *atg5*-up GACATCCAACCGCTCTGCGCA, *atg5*-down CAGACGATGACTTCACGTACACC, *act5C*-F CACACCAAATCTTACAAAATGTGTGA, *act5C*-R AATCCGGCCTTGCACATG. Primers were optimized (Advanced Biosystems procedure), and relative quantities determined (relative standard curve method) and normalized to *actin5C*.

Paraquat injections

Injection pipettes were drawn out of 10mm glass capillaries using a Flaming-Brown micropipette puller (Programme 2). A home-built microinjection machine was used to deliver paraquat at a final dose of 50ng/mg. Both paraquat solution and mock control solution contained blue dye for visualization of injections (FD&C Blue No.1).

Feeding assay

Flies were reared as for the survival analysis and placed at 5 per vial with no anaesthesia 24 hours prior to observation. Feeding assays were carried out by direct observation on undisturbed flies (Wong et al., 2008). The number of feeding events, as measured by proboscis extension into solid medium, was recorded. Data is presented as the proportion of flies feeding during the observation period.

In order to measure the amount of food that was consumed by flies and to determine if there was a correlation between observed feeding behavior and food intake, we have performed dye-calibrated feeding observation as described in (Wong et al., 2009). Briefly, 7 day-old female flies were put on standard SYA food containing 2.5% blue dye (w/w; FD&C Blue No.1) and 200 µM rapamycin or ethanol as a control. Feeding was observed for 30 minutes, and flies were then frozen in liquid nitrogen. The amount of blue dye was determined spectrophotometrically. The relationship between observed feeding events and blue-dye consumption was analyzed as previously described (Wong et al., 2009).

Lysotracker staining, imaging and image analysis

Lysotracker staining is based on selective visualisation of lysosomes and autophagosomes due to their low pH and is a reliable steady state method that gives an indication of autophagy induction. For Lysotracker staining, complete guts were removed from flies that had been maintained on 200 µM rapamycin or control food for 5 days (N = 5 flies in each group). Dissections were performed in PBS. Each gut was mounted into a custom made imaging chamber and stained with Lysotracker DS Red DND-99 (Invitrogen, Molecular Probes,) 1 µM for 3min. Each preparation was then washed three times with PBS and mounted in mounting medium (Mounting medium, Vectashield, H1200) containing DAPI (1.5µg/ml). Imaging was performed using a Zeiss LSM 700 confocal microscope with a 20x objective. In each preparation an area of midgut proximal to the proventriculus was imaged to control for variation along the gut. Three separate, adjacent 100 mm^2 images were obtained from each preparation. Laser power (at 555 nm), digital gain and optical settings were kept constant between the images. Each image was then background subtracted and passed through a high pass digital filter (Lucida Image Analysis, Kinetic Imaging, Liverpool, UK) to enhance punctate structures. Images were then thresholded to binarise and quantify pixels of high value. The mean sum of these pixels for each condition is presented.

Mass Spectroscopy analysis for quantification of rapamycin in flies

Mass Spectrometry measurements were performed in order to determine the concentration of rapamycin in flies, using methods adapted from (Serkova et al., 1999).

15 flies per sample were homogenised in 500 µl of 1 M potassium phosphate buffer, pH 7.5. After homogenization, all samples were spiked with the 0.1 nmoles everolimus (LC Laboratories) as an internal standard. For the standard curve, non-rapamycin treated flies were spiked with various rapamycin concentrations ranging from 0.1 pmoles to 0.1 µmoles rapamycin. Proteins were precipitated by the addition of 1mL of methanol/1M $ZnSO_4$ (80:20, v/v). After vortexing and centrifugation (5 minutes at 20,000 *g*), supernatant was loaded onto a C_{18} extraction column (Sep-Pak WAT 054945) by vacuum. Rapamycin and everolimus were then co-eluted with 1.5 mL methylene chloride into clean tubes. Samples were dried using a speed-vacuum. Samples were resuspended in 100 µl acetonitrile/water (pH 3; 75:25; v/v). Mass spectrometry analysis of rapamycin was performed by Dr. Carolyn Hyde of the Scientific Support Services, Wolfson Institute for Biomedical Research, University College London. Rapamycin samples (10 µl) were run on Shimadzu LC-MS-ITToF.

^{35}S methionine incorporation.

Standard sugar-yeast-agar based food was supplemented with 100 µCi^{35}S-methionine/ml of food (American Radiolabeled Chemicals 1mCi/37MBq ARS 0104A). 15 flies were transferred to each vial containing radioactive food. After 3h of feeding flies were transferred to non-radioactive food for 30min in order to purge undigested ^{35}S-methionine radioactive food out of the intestines. Flies that were in contact with the radioactive food for 1 minute were used as a background control. Flies were then homogenized in 200 µM 1% SDS and heated for 5 minutes at 95˚C. Samples were centrifuged for 2x5 minutes at maximum speed and supernatant retained. Proteins were precipitated by the addition of the same volume of 20% cold TCA (10% TCA final concentration) and incubated for 15 minutes on ice. Samples were then centrifuged at 16,000 *g* for 15 minutes, the pellet washed twice with acetone and then resuspended in 200 µl of 4M guanidine-HCl. Samples (100 µl) were mixed with 3 ml of scintillant (Fluoran-Safe 2, BDH) and radioactivity counted in a liquid scintillation analyzer (Tri-Carb 2800TR, Perkin Elmer), with appropriate quench corrections. Measurements were normalized to total protein for each sample, as determined using Bradford reagent (BioRad).

Supplemental References

Bass, T.M., Grandison, R.C., Wong, R., Martinez, P., Partridge, L., and Piper, M.D. (2007). Optimization of dietary restriction protocols in Drosophila. J Gerontol A Biol Sci Med Sci 62, 1071-1081.

Bohni, R., Riesgo-Escovar, J., Oldham, S., Brogiolo, W., Stocker, H., Andruss, B.F., Beckingham, K., and Hafen, E. (1999). Autonomous control of cell and organ size by CHICO, a Drosophila homolog of vertebrate IRS1-4. Cell 97, 865-875.

Gronke, S., Beller, M., Fellert, S., Ramakrishnan, H., Jackle, H., and Kuhnlein, R.P. (2003). Control of fat storage by a Drosophila PAT domain protein. Curr Biol 13, 603-606.

Ikeya, T., Galic, M., Belawat, P., Nairz, K., and Hafen, E. (2002). Nutrient-dependent expression of insulin-like peptides from neuroendocrine cells in the CNS contributes to growth regulation in Drosophila. Curr Biol 12, 1293-1300.

Ren, C., Finkel, S.E., and Tower, J. (2009). Conditional inhibition of autophagy genes in adult Drosophila impairs immunity without compromising longevity. Exp Gerontol 44, 228-235.

Scott, R.C., Schuldiner, O., and Neufeld, T.P. (2004). Role and regulation of starvation-induced autophagy in the Drosophila fat body. Dev Cell 7, 167-178.

Serkova, N., Litt, L., James, T.L., Sadee, W., Leibfritz, D., Benet, L.Z., and Christians, U. (1999). Evaluation of individual and combined neurotoxicity of the immunosuppressants cyclosporine and sirolimus by in vitro multinuclear NMR spectroscopy. J Pharmacol Exp Ther 289, 800-806.

Tettweiler, G., Miron, M., Jenkins, M., Sonenberg, N., and Lasko, P.F. (2005). Starvation and oxidative stress resistance in Drosophila are mediated through the eIF4E-binding protein, d4E-BP. Genes Dev 19, 1840-1843.

Toivonen, J.M., Walker, G.A., Martinez-Diaz, P., Bjedov, I., Driege, Y., Jacobs, H.T., Gems, D., and Partridge, L. (2007). No influence of Indy on lifespan in Drosophila after correction for genetic and cytoplasmic background effects. PLoS Genet 3, e95.

Wong, R., Piper, M.D., Blanc, E., and Partridge, L. (2008). Pitfalls of measuring feeding rate in the fruit fly Drosophila melanogaster. Nat Methods 5, 214-215; author reply 215.

Wong, R., Piper, M.D., Wertheim, B., and Partridge, L. (2009). Quantification of food intake in Drosophila. PLoS One 4, e6063.
